# Hospitalizations for Inflammatory Bowel Disease Among Medicare Fee-for-Service Beneficiaries — United States, 1999–2017

**DOI:** 10.15585/mmwr.mm6849a2

**Published:** 2019-12-13

**Authors:** Fang Xu, Anne G. Wheaton, Yong Liu, Hua Lu, Kurt J. Greenlund

**Affiliations:** 1 Division of Population Health, National Center for Chronic Disease Prevention and Health Promotion, CDC.

Crohn’s disease and ulcerative colitis, collectively referred to as inflammatory bowel disease (IBD), are conditions characterized by chronic inflammation of the gastrointestinal tract. The incidence and prevalence of IBD is increasing globally, and although the disease has little impact on mortality, the number of older adults with IBD is expected to increase as the U.S. population ages (*1*). Older adults with IBD have worse hospitalization outcomes than do their younger counterparts (*2*). CDC analyzed Medicare Provider Analysis and Review (MedPAR) data to estimate IBD-related hospitalization rates and hospitalization outcomes in 2017 among Medicare fee-for-service beneficiaries aged ≥65 years, by selected demographics and trends in hospitalization rates and by race/ethnicity during 1999–2017. In 2017, the age-adjusted hospitalization rate for Crohn’s disease was 15.5 per 100,000 Medicare enrollees, and the IBD-associated surgery rate was 17.4 per 100 hospital stays. The age-adjusted hospitalization rate for ulcerative colitis was 16.2 per 100,000 Medicare enrollees, and the surgery rate was 11.2 per 100 stays. During 1999–2017, the hospitalization rate for both Crohn’s disease and ulcerative colitis decreased among non-Hispanic white (white) beneficiaries, but not among non-Hispanic black (black) beneficiaries. Health care utilization assessment is needed among black beneficiaries with IBD. Disease management for older adults with IBD could focus on increasing preventive care and preventing emergency surgeries that might result in further complications.

MedPAR data, obtained from the Centers for Medicare & Medicaid Services, contain information on 100% of Medicare beneficiaries who use hospital inpatient services and skilled nursing facilities, including information about admission, discharge, diagnosis, and procedure codes related to a hospital stay.* To estimate IBD-related hospitalization rates and hospitalization outcomes in 2017, IBD-related hospital admissions^†^ based on *International Classification of Diseases, Tenth Edition,* *Clinical Modification* (ICD-10-CM) principal diagnosis codes (K50 for Crohn’s disease and K51 for ulcerative colitis) were used to identify eligible beneficiaries from 50 states and the District of Columbia (DC) who were aged ≥65 years at the time of Medicare enrollment and hospital admission, were continuously enrolled in Medicare Part A, and were not enrolled in a Health Maintenance Organization in 2017. To analyze temporal trends in hospitalization rates by race/ethnicity, ICD-9-CM diagnosis codes (555 for Crohn’s disease and 556 for ulcerative colitis) were used for hospitalizations before October 1, 2015, and ICD-10-CM codes for those on or after that date. The age-adjusted hospitalization rate (hereafter referred to as hospitalization rate) was estimated per 100,000 eligible Medicare enrollees^§^ for 50 states and DC and by selected demographic characteristics. The surgery rate was defined as the number of partial or total resections of the small or large intestine per 100 hospital stays.^¶^ The 30-day readmission rate was calculated as the number of all-cause readmissions to acute-care, nonfederal hospitals within 30 days of the discharge date associated with the index admission, per 100 hospital stays.** The 30-day mortality rate was defined as the number of all-cause deaths that occurred within 30 days of the index admission per 100 hospital stays.^††^ All hospitalization outcome rates were calculated with 95% confidence intervals by demographic variables including age group (65–74 and ≥75 years), sex, race/ethnicity (white, black, other [Hispanic, Asian/Pacific Islander, American Indian/Alaska Native, and other], or unknown)^§§^ and urban or rural residence of beneficiary.^¶¶^ Group differences were determined by Z-test with the significance level set at alpha = 0.05. Trends in hospitalization rates were assessed using a linear regression. All analyses were performed separately for Crohn’s disease and ulcerative colitis using SAS Enterprise Guide (version 7.1; SAS Institute).

In 2017, among approximately 30,658,000 eligible Medicare enrollees aged ≥65 years, 4,782 hospital admissions were attributable to Crohn’s disease (15.5 per 100,000 Medicare population), and 4,932 were attributable to ulcerative colitis (16.2 per 100,000 Medicare population) (Table). For both diseases, the hospitalization rate was higher among women, whites, and urban residents than among men, blacks, and rural residents. Compared with older beneficiaries (those aged ≥75 years), younger beneficiaries (those aged 65–74 years) had a higher rate of hospitalization for Crohn’s disease but a lower rate for ulcerative colitis. Overall, the surgery rate was 17.4 per 100 hospital stays for Crohn’s disease and 11.2 per 100 stays for ulcerative colitis. For both diseases, the surgery rate was lower among older beneficiaries, women, and blacks than among their counterparts. The 30-day readmission rate for ulcerative colitis was higher among older beneficiaries, and for Crohn’s disease was higher among men than women. The 30-day mortality rates for Crohn’s disease and ulcerative colitis were 2.7 and 3.8 per 100 stays, respectively. For both diseases, the 30-day mortality rate was higher among older beneficiaries. The hospitalization rate was generally higher in the Midwest for Crohn’s disease and in the Northeast for ulcerative colitis (Figure 1). In each year during 1999–2017 a modest decrease occurred in the hospitalization rate for Crohn’s disease overall (0.08 per 100,000 eligible Medicare enrollees, p = 0.002) and among whites (0.07, p = 0.01), but no significant change among blacks occurred (p = 0.11) (Figure 2). In each year during 1999–2017, the hospitalization rate for ulcerative colitis decreased overall (0.31, p<0.001) and among whites (0.32, p<0.001), with no significant change among blacks (p = 0.06).

**TABLE:** Hospitalizations for inflammatory bowel disease (IBD)* as the principal diagnosis among Medicare fee-for-service beneficiaries,^†^ by age, sex, race/ethnicity, and urban-rural status of residence — United States, 2017

Characteristic	No. of hospitalizations	Hospitalization rate,^§^ (95% CI)	Surgery rate, ^¶,^**^,††^ (95% CI)	30-Day readmission rate,^¶,§§^ (95% CI)	30-Day mortality rate,^¶,¶¶^ (95% CI)	Length of stay (days)** geometric mean,*** (95% CI)
**Crohn’s disease^†††^**						
**Total**	**4,782**	**15.5 (15.0–15.9)**	**17.4 (16.3–18.4)**	**15.8 (14.5–17.1)**	**2.7 (2.2–3.1)**	**3.9 (3.8–4.0)**
**Age group (yrs)**						
65–74	2,912	17.1 (16.5–17.7)	19.3 (17.8–20.7)	15.4 (13.7–17.0)	1.7 (1.2–2.1)	3.8 (3.7–3.9)
≥75	1,870	13.7 (13.1–14.4)	14.4 (12.8–16.0)	16.5 (14.3–18.7)	4.2 (3.3–5.1)	4.1 (3.9–4.2)
**Sex**						
Men	2,001	14.1 (13.5–14.8)	19.0 (17.3–20.8)	17.6 (15.5–19.7)	3.0 (2.3–3.8)	3.8 (3.6–3.9)
Women	2,781	16.7 (16.1–17.3)	16.1 (14.8–17.5)	14.5 (12.7–16.1)	2.4 (1.8–3.0)	4.0 (3.9–4.1)
**Race/Ethnicity**						
White, non-Hispanic	4,227	16.9 (16.4–17.5)	17.5 (16.4–18.7)	16.1 (14.6–17.5)	2.6 (2.1–3.0)	4.0 (3.9–4.0)
Black, non-Hispanic	250	10.3 (9.0–11.5)	10.8 (7.0–14.6)	14.7 (9.1–20.2)	—^§§§^	4.2 (4.0–4.5)
Other or unknown^¶¶¶^	305	8.8 (7.8–9.8)	20.3 (15.8–24.8)	13.1 (8.3–17.7)	—^§§§^	4.1 (3.8–4.3)
Beneficiary residence****						
Urban	3,938	16.0 (15.5–16.5)	17.0 (15.8–18.2)	16.1 (14.6–17.5)	2.6 (2.1–3.1)	3.9 (3.8–4.0)
Rural	844	13.4 (12.5–14.3)	19.0 (16.3–21.6)	14.5 (11.3–17.7)	3.1 (1.9–4.3)	3.8 (3.7–4.0)
**Ulcerative colitis^†††^**						
**Total**	**4,932**	**16.2 (15.8**–**16.7)**	**11.2 (10.3**–**12.1)**	**16.0 (14.6**–**17.3)**	**3.8 (3.3**–**4.4)**	**4.1 (4.0**–**4.2)**
**Age group (yrs)**						
65–74	2,395	14.1 (13.5–14.6)	15.6 (14.1–17.0)	14.5 (12.7–16.3)	2.1 (1.5–2.7)	4.1 (4.0–4.3)
≥75	2,537	18.6 (17.9–19.4)	7.0 (6.0–8.0)	17.4 (15.5–19.3)	5.5 (4.6–6.4)	4.0 (3.9–4.2)
**Sex**						
Men	1,872	13.5 (12.9–14.2)	16.9 (15.2–18.6)	17.6 (15.3–19.8)	4.0 (3.1–4.9)	4.2 (4.1–4.4)
Women	3,060	18.4 (17.7–19.0)	7.6 (6.7–8.6)	15.0 (13.4–16.6)	3.7 (3.1–4.4)	4.0 (3.9–4.1)
**Race/Ethnicity**						
White, non-Hispanic	4,205	17.0 (16.5–17.5)	11.5 (10.6–12.5)	16.0 (14.6–17.4)	4.0 (3.4–4.6)	4.0 (3.9–4.0)
Black, non-Hispanic	325	14.0 (12.4–15.5)	7.1 (4.3–9.9)	18.9 (13.5–24.1)	—^§§§^	4.2 (4.0–4.5)
Other or unknown^¶¶¶^	402	12.0 (10.8–13.3)	10.7 (7.7–13.7)	13.2 (8.9–17.4)	—^§§§^	4.1 (3.8–4.3)
Beneficiary residence****						
Urban	4,110	17.0 (16.5–17.6)	10.8 (9.9–11.8)	16.2 (14.7–17.6)	3.9 (3.3–4.5)	4.1 (4.0–4.2)
Rural	819	13.2 (12.2–14.1)	12.9 (10.6–15.2)	14.9 (11.5–18.1)	3.4 (2.2–4.7)	4.0 (3.8–4.2)

**Abbreviation:** CI = confidence interval.

* IBD encompasses Crohn’s disease and ulcerative colitis.

^†^ The number of eligible Medicare beneficiaries aged ≥65 years in 2017 in the United States was approximately 30,658,000.

^§^ Hospitalizations per 100,000 Medicare enrollees, age-adjusted to the 2000 U.S. Standard Population aged ≥65 years based on two age groups (65–74 years and ≥75 years), except for age-specific analysis. https://www.cdc.gov/nchs/data/statnt/statnt20.pdf.

^¶^ Per 100 hospital stays.

** The denominator is number of hospital admissions. To calculate surgery rate and geometric mean of length of stay, n = 4,782 for Crohn’s disease and n = 4,932 for ulcerative colitis.

^††^ *International Classification of Diseases, Tenth Edition, Procedure Coding System* procedure codes involving small and large intestine include partial resection (0DB80ZZ, 0DB90ZZ, 0DBA0ZZ, 0DBB0ZZ, 0DBC0ZZ, 0DBE0ZZ, 0DBF0ZZ, 0DBG0ZZ, 0DBH0ZZ, 0DBK0ZZ, 0DBL0ZZ, 0DBM0ZZ, 0DBN0ZZ, 0DBP0ZZ, 0DB83ZZ, 0DB93ZZ, 0DBA3ZZ, 0DBB3ZZ, 0DBC3ZZ, 0DBE3ZZ, 0DBF3ZZ, 0DBG3ZZ, 0DBH3ZZ, 0DBK3ZZ, 0DBL3ZZ, 0DBM3ZZ, 0DBN3ZZ, 0DBP3ZZ, 0DB84ZZ, 0DB94ZZ, 0DBA4ZZ, 0DBB4ZZ, 0DBC4ZZ, 0DBE4ZZ, 0DBF4ZZ, 0DBG4ZZ, 0DBH4ZZ, 0DBK4ZZ, 0DBL4ZZ, 0DBM4ZZ, 0DBN4ZZ, 0DBP4ZZ, 0DB87ZZ, 0DB97ZZ, 0DBA7ZZ, 0DBB7ZZ, 0DBC7ZZ, 0DBE7ZZ, 0DBF7ZZ, 0DBG7ZZ, 0DBH7ZZ, 0DBK7ZZ, 0DBL7ZZ, 0DBM7ZZ, 0DBN7ZZ, 0DBP7ZZ, 0DB88ZZ, 0DB98ZZ, 0DBA8ZZ, 0DBB8ZZ, 0DBC8ZZ, 0DBE8ZZ, 0DBF8ZZ, 0DBG8ZZ, 0DBH8ZZ, 0DBK8ZZ, 0DBL8ZZ, 0DBM8ZZ, 0DBN8ZZ, 0DBP8ZZ) and total resection (0DT80ZZ, 0DT90ZZ, 0DTA0ZZ, 0DTB0ZZ, 0DTC0ZZ, 0DTE0ZZ, 0DTF0ZZ, 0DTG0ZZ, 0DTH0ZZ, 0DTK0ZZ, 0DTL0ZZ, 0DTM0ZZ, 0DTN0ZZ, 0DTP0ZZ, 0DT84ZZ, 0DT94ZZ, 0DTA4ZZ, 0DTB4ZZ, 0DTC4ZZ, 0DTE4ZZ, 0DTF4ZZ, 0DTG4ZZ, 0DTH4ZZ, 0DTK4ZZ, 0DTL4ZZ, 0DTM4ZZ, 0DTN4ZZ, 0DTP4ZZ, 0DT87ZZ, 0DT97ZZ, 0DTA7ZZ, 0DTB7ZZ, 0DTC7ZZ, 0DTE7ZZ, 0DTF7ZZ, 0DTG7ZZ, 0DTH7ZZ, 0DTK7ZZ, 0DTL7ZZ, 0DTM7ZZ, 0DTN7ZZ, 0DTP7ZZ, 0DT88ZZ, 0DT98ZZ, 0DTA8ZZ, 0DTB8ZZ, 0DTC8ZZ, 0DTE8ZZ, 0DTF8ZZ, 0DTG8ZZ, 0DTH8ZZ, 0DTK8ZZ, 0DTL8ZZ, 0DTM8ZZ, 0DTN8ZZ, 0DTP8ZZ).

^§§^ 30-day readmission rate was calculated as the number of all-cause readmissions to acute care, nonfederal hospitals within 30 days from the discharge date associated with an index admission per 100 stays. Index admissions were nonfederal acute-care hospital admissions with Crohn’s disease or ulcerative colitis as the principal diagnosis, had a live discharge, and had a routine discharge to home or skilled nursing facility. To ensure the calculation of the complete 30-day readmission, index admissions with discharge date in December 2017 were excluded. Readmissions were defined as all-cause acute admissions whose discharge destinations were not against medical advice or expired. Multiple readmissions within the 30-day time frame from one index admission were counted as one readmission. A readmission from a previous index admission could be also counted as a new index admission if it met the selection criteria for an index admission (https://www.medicare.gov/hospitalcompare/data/30-day-measures.html). To calculate 30-day all-cause readmission rate, n = 3,022 for Crohn’s disease; n = 3,060 for ulcerative colitis.

^¶¶^ 30-day mortality rate was defined as number of all-cause deaths occurred within 30 days from IBD-related admissions per 100 hospital stays. Hospitalizations were excluded if patients were discharged against medical advice. More information is available at https://www.medicare.gov/hospitalcompare/data/30-day-measures.html. To calculate 30-day all-cause readmission rate, n = 4,781 for Crohn’s disease; n = 4,932 for ulcerative colitis.

*** Geometric mean is the x^th^ root of the product of the length of stay (days) from x patients (x indicates the number of patients), which, because it is not influenced by outliers, is used here rather than arithmetic mean.

^†††^ *International Classification of Diseases, Tenth Edition, Clinical Modification* diagnosis codes K50 (Crohn’s disease) and K51 (ulcerative colitis).

^§§§^ Data were suppressed according to the cell size suppression (https://www.resdac.org/articles/cms-cell-size-suppression-policy) or if the relative standard error was >0.3.

^¶¶¶^ Other includes Hispanic, Asian, Native American, and all others.

**** Urban areas include large metro, large fringe metro, medium metro, or small metro. Rural areas include micropolitan and noncore. The definition is based on 2013 National Center for Health Statistics Urban-Rural Classification Scheme for Counties (https://www.cdc.gov/nchs/data/series/sr_02/sr02_166.pdf).

**FIGURE 1:**
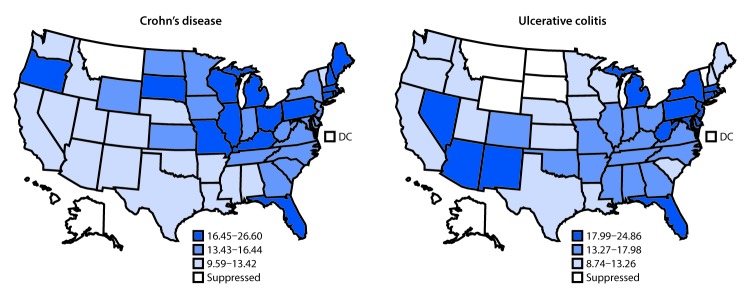
Age-adjusted hospitalization rates*^,†^ for Crohn’s disease or ulcerative colitis^§^ as the principal diagnosis among Medicare fee-for-service beneficiaries, by state — United States, 2017 **Abbreviation:** DC = District of Columbia. * Hospitalizations per 100,000 eligible Medicare enrollees, age-adjusted to the 2000 U.S. standard population aged ≥65 years (https://www.cdc.gov/nchs/data/statnt/statnt20.pdf) based on two age groups (65–74 years and ≥75 years). ^†^ State-level age-adjusted hospitalization rates were categorized into tertiles. Data were suppressed if relative standard errors were >0.3. ^§^ Crohn’s disease and ulcerative colitis, collectively referred to as inflammatory bowel disease, are conditions characterized by chronic inflammation of the gastrointestinal tract.

**FIGURE 2:**
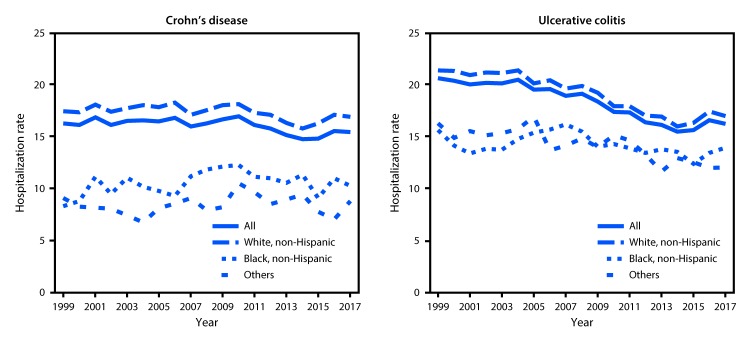
Age-adjusted hospitalization rates*^,†^ for inflammatory bowel disease**^§^** as the principal diagnosis among Medicare fee-for-service beneficiaries, by race/ethnicity — United States, 1999–2017**^¶^** * Hospitalizations per 100,000 eligible Medicare enrollees, age adjusted to the 2000 U.S. standard population aged ≥65 years (https://www.cdc.gov/nchs/data/statnt/statnt20.pdf) based on two age groups (65–74 years and ≥75 years). ^†^ Linear trend p-values were assessed from a linear regression. ^§^ Inflammatory bowel disease is a term for two conditions (Crohn’s disease and ulcerative colitis) that are characterized by chronic inflammation of the gastrointestinal tract. ^¶^ The conversion from *International Classification of Diseases, Ninth Edition* diagnosis codes to the *International Classification of Diseases, Tenth Edition, Clinical Modification* diagnosis codes occurred on October 1, 2015.

## Discussion

During 1999–2017, the overall hospitalization rate for both Crohn’s disease and ulcerative colitis decreased among older adults, with a sharper decline in the hospitalization rate for ulcerative colitis. A previous study also reported that the 2013 hospitalization rate for Crohn’s disease decreased compared with that in 2003 among adults aged 65–84 and ≥85 years (*3*). The overall decline in hospitalization rates during the current study period was accompanied by the evolution of biologic therapies to treat IBD. A unique geographic pattern of hospitalization rates at the state level was observed for each disease. The geographic variation was similar to that in the previous study, which used the Nationwide Inpatient Sample of adults aged ≥18 years with hospitalizations for any listed diagnosis of Crohn’s disease (*3*). In addition, the IBD-related hospitalization rate was higher among beneficiaries who were urban residents than among those who were rural residents. An urban living environment was previously found to be associated with a higher risk of developing IBD, although rural residents might also have limited health care access to receive IBD-related care or diagnosis (*4*).

The current study indicated that hospitalization rates were higher among older Medicare beneficiaries with ulcerative colitis and among younger beneficiaries with Crohn’s disease; however, the rate of IBD-associated surgery was lower among older beneficiaries for both conditions. Surgery is indicated in severe cases or for patients who fail to respond to medication. Although the overall IBD-associated surgical rates have declined in recent decades, possibly because of new medication treatment (*5*), in the current analysis, approximately 10% of hospitalizations for ulcerative colitis and nearly 20% of hospitalizations for Crohn’s disease still required surgery. The lower surgery rates among older beneficiaries might reflect an increased concern for postoperative complications, mortality, prolonged hospital stay, and hospital-acquired infections among older patients (*2*).

This is the first study to document the temporal trends in IBD-associated hospitalization during the past 2 decades among older adults in United States by race/ethnicity. In contrast to the decreased hospitalization rates for both diseases observed among whites, no significant temporal changes in hospitalization rates among blacks were observed. In addition, in 2017, blacks experienced lower hospitalization rates for both Crohn’s disease and ulcerative colitis than did whites. These findings are consistent with those in a previous study in which reported rates of IBD incidence, prevalence, and hospitalization in blacks and Hispanics were lower than were those reported for whites (*6*). However, this previous study also found that the ratio of IBD-associated hospitalizations and mortality to IBD prevalence was higher among blacks than that among whites and Hispanics (*6*). Another study found lower use of biologics or lower adherence to medications among blacks (*7*), which could contribute to the higher ratio of IBD hospitalization to IBD prevalence among this group.

The findings in the report are subject to at least three limitations. First, Medicare data are collected for insurance reimbursement purposes and are not designed for research. The collected data do not include information about health-risk behaviors and additional demographic variables, which limited the ability to study these measures. Second, diagnoses or procedures might be subject to coding errors. Finally, the study population is limited to Medicare fee-for-service beneficiaries. Therefore, the findings might not be generalizable to all U.S. adults aged ≥65 years.

IBD management is challenging because comorbidities and polypharmacy are common among older adults. For older adults, the necessity of surgery should be carefully evaluated based on an individual patient’s disease severity and comorbid mental and physical conditions (*8*). If surgery is indicated and performed, early intervention, together with pain control and a proper discharge plan might prevent poor hospitalization outcomes, such as readmissions, and might ultimately reduce health care costs (*9*). In addition, further assessment of health care utilization among blacks with IBD is needed. Optimal multidisciplinary disease management, including outpatient follow-up visits and receiving recommended preventive care such as vaccinations and cancer screening (*10*), is important to maintain remission, improve quality of life, and prevent surgery and hospitalization among the growing population of older adults with IBD.

**Summary**

**What is already known about this topic?**

The number of older adults with Crohn’s disease or ulcerative colitis, collectively referred to as inflammatory bowel disease (IBD), is expected to increase as the U.S. population ages.

**What is added by this report?**

In 2017, the hospitalization rates for Crohn’s disease and ulcerative colitis (approximately 16 hospitalizations per 100,000 Medicare beneficiaries) were higher among urban than rural beneficiaries. Surgery rates for Crohn’s disease and ulcerative colitis were 17 and 11 per 100 stays, respectively. During 1999–2017, hospitalization rates for IBD decreased among whites but not among blacks.

**What are the implications for public health practice?**

Disease management among older adults with IBD could focus on achieving and maintaining remission and preventing IBD-related emergency surgery.

## Footnotes

* https://www.cms.gov/Research-Statistics-Data-and-Systems/Statistics-Trends-and-Reports/MedicareFeeforSvcPartsAB/MEDPAR.

† Hospital admissions were limited to those at short-stay facilities. https://www.resdac.org/cms-data/variables/medpar-short-staylong-staysnf-indicator-code.

§ Direct age adjustment according to 2000 U.S. Standard Population based on two age groups (65–74 years and ≥75 years). https://www.cdc.gov/nchs/data/statnt/statnt20.pdf.

¶ Surgery was identified using *International Classification of Diseases, Tenth Edition, Procedure Coding System* involving small or large intestines. Partial resection: 0DB80ZZ, 0DB90ZZ, 0DBA0ZZ, 0DBB0ZZ, 0DBC0ZZ, 0DBE0ZZ, 0DBF0ZZ, 0DBG0ZZ, 0DBH0ZZ, 0DBK0ZZ, 0DBL0ZZ, 0DBM0ZZ, 0DBN0ZZ, 0DBP0ZZ, 0DB83ZZ, 0DB93ZZ, 0DBA3ZZ, 0DBB3ZZ, 0DBC3ZZ, 0DBE3ZZ, 0DBF3ZZ, 0DBG3ZZ, 0DBH3ZZ, 0DBK3ZZ, 0DBL3ZZ, 0DBM3ZZ, 0DBN3ZZ, 0DBP3ZZ, 0DB84ZZ, 0DB94ZZ, 0DBA4ZZ, 0DBB4ZZ, 0DBC4ZZ, 0DBE4ZZ, 0DBF4ZZ, 0DBG4ZZ, 0DBH4ZZ, 0DBK4ZZ, 0DBL4ZZ, 0DBM4ZZ, 0DBN4ZZ, 0DBP4ZZ, 0DB87ZZ, 0DB97ZZ, 0DBA7ZZ, 0DBB7ZZ, 0DBC7ZZ, 0DBE7ZZ, 0DBF7ZZ, 0DBG7ZZ, 0DBH7ZZ, 0DBK7ZZ, 0DBL7ZZ, 0DBM7ZZ, 0DBN7ZZ, 0DBP7ZZ, 0DB88ZZ, 0DB98ZZ, 0DBA8ZZ, 0DBB8ZZ, 0DBC8ZZ, 0DBE8ZZ, 0DBF8ZZ, 0DBG8ZZ, 0DBH8ZZ, 0DBK8ZZ, 0DBL8ZZ, 0DBM8ZZ, 0DBN8ZZ, 0DBP8ZZ; Total resection: 0DT80ZZ, 0DT90ZZ, 0DTA0ZZ, 0DTB0ZZ, 0DTC0ZZ, 0DTE0ZZ, 0DTF0ZZ, 0DTG0ZZ, 0DTH0ZZ, 0DTK0ZZ, 0DTL0ZZ, 0DTM0ZZ, 0DTN0ZZ, 0DTP0ZZ, 0DT84ZZ, 0DT94ZZ, 0DTA4ZZ, 0DTB4ZZ, 0DTC4ZZ, 0DTE4ZZ, 0DTF4ZZ, 0DTG4ZZ, 0DTH4ZZ, 0DTK4ZZ, 0DTL4ZZ, 0DTM4ZZ, 0DTN4ZZ, 0DTP4ZZ, 0DT87ZZ, 0DT97ZZ, 0DTA7ZZ, 0DTB7ZZ, 0DTC7ZZ, 0DTE7ZZ, 0DTF7ZZ, 0DTG7ZZ, 0DTH7ZZ, 0DTK7ZZ, 0DTL7ZZ, 0DTM7ZZ, 0DTN7ZZ, 0DTP7ZZ, 0DT88ZZ, 0DT98ZZ, 0DTA8ZZ, 0DTB8ZZ, 0DTC8ZZ, 0DTE8ZZ, 0DTF8ZZ, 0DTG8ZZ, 0DTH8ZZ, 0DTK8ZZ, 0DTL8ZZ, 0DTM8ZZ, 0DTN8ZZ, 0DTP8ZZ.

** Index admissions were nonfederal, acute-care hospital admissions with Crohn’s disease or ulcerative colitis as the principal diagnosis, had a live discharge, and had a routine discharge to home or skilled nursing facility. To ensure the calculation of the complete 30-day readmission, index admissions with discharge date in December 2017 were excluded. Readmissions were defined as all-cause acute admissions in which discharge destinations for the index admission were not against medical advice or expired. Multiple readmissions within the 30-day time frame from one index admission were counted as one readmission. A readmission from a previous index admission could be also be counted as a new index admission if it met the selection criteria for an index admission. https://www.medicare.gov/hospitalcompare/data/30-day-measures.html.

†† Hospital admissions were excluded if patients were discharged against medical advice. https://www.medicare.gov/hospitalcompare/data/30-day-measures.html.

§§ https://www.resdac.org/cms-data/variables/research-triangle-institute-rti-race-code.

¶¶ Based on 2013 National Center for Health Statistics Urban-Rural Classification Scheme for Counties. Urban counties include large metro, large fringe metro, medium metro, or small metro. Rural counties include micropolitan and noncore. https://www.cdc.gov/nchs/data/series/sr_02/sr02_166.pdf.
